# A Polar Initial Alignment Algorithm for Unmanned Underwater Vehicles

**DOI:** 10.3390/s17122709

**Published:** 2017-11-23

**Authors:** Zheping Yan, Lu Wang, Tongda Wang, Honghan Zhang, Xun Zhang, Xiangling Liu

**Affiliations:** College of Automation, Harbin Engineering University, Harbin 150001, China; yanzheping@hrbeu.edu.cn (Z.Y.); wangtongda@hrbeu.edu.cn (T.W.); zhanghonghan2008@163.com (H.Z.); zhangxun2008@hrbeu.edu.cn (X.Z.); liuxiangling0229@163.com (X.L.)

**Keywords:** unmanned underwater vehicle, initial alignment, grid frame, polar region

## Abstract

Due to its highly autonomy, the strapdown inertial navigation system (SINS) is widely used in unmanned underwater vehicles (UUV) navigation. Initial alignment is crucial because the initial alignment results will be used as the initial SINS value, which might affect the subsequent SINS results. Due to the rapid convergence of Earth meridians, there is a calculation overflow in conventional initial alignment algorithms, making conventional initial algorithms are invalid for polar UUV navigation. To overcome these problems, a polar initial alignment algorithm for UUV is proposed in this paper, which consists of coarse and fine alignment algorithms. Based on the principle of the conical slow drift of gravity, the coarse alignment algorithm is derived under the grid frame. By choosing the velocity and attitude as the measurement, the fine alignment with the Kalman filter (KF) is derived under the grid frame. Simulation and experiment are realized among polar, conventional and transversal initial alignment algorithms for polar UUV navigation. Results demonstrate that the proposed polar initial alignment algorithm can complete the initial alignment of UUV in the polar region rapidly and accurately.

## 1. Introduction

In recent years, the development of unmanned underwater vehicles (UUVs) has accelerated notably. UUVs play important roles in many aspects [1,2]. UUVs can accomplish missions in harsh environments, for example, the polar region. Strapdown inertial navigation systems (SINSs) are widely used in UUVs both in the polar region and in non-polar regions since they are highly autonomous [3]. A SINS is essentially a process of recursive calculation under some initial conditions. It is based on the output of inertial measurement units (IMUs) that consists of gyroscopes and accelerometers. Initial alignment is crucial because the initial alignment results will be used as the initial SINS value, which might affect the subsequent SINS results [4,5]. The initial position and initial velocity can be obtained with the help of external auxiliary information. The main task of initial alignment is to determine the accurate initial attitude matrix. Initial alignment of SINS in UUV is intended to determine the initial attitude matrix in a short time with certain accuracy [6]. Global navigation satellite systems, such as Global Position System (GPS), GLONASS, Galileo and Compass can provide an accurate reference position. A Doppler Velocity Log (DVL) can provide an accurate reference velocity. Therefore, the initial position and initial velocity can be easily determined. Especially for a temporarily anchored UUV in the polar region considered in this paper, the initial velocity is zero. To simplify the analysis and for the purposes of this paper, the initial position and initial velocity are assumed to be well provided. This polar initial alignment algorithm mainly focuses on the determination of the initial attitude of UUV in the polar region. There are two important indicators to measure the performance of an initial alignment algorithm, which are alignment accuracy and alignment speed [7]. It is our goal to complete the initial alignment rapidly and accurately. Based on the motion characteristics of the base, initial alignments can be categorized into three types: the stationary base, the rocking base and the moving base. In the process of initial alignment, UUV is interfered by ocean waves. The IMU output contains this interference error. Bigger waves will result in more serious interference errors and lower signal-to noise ratios, making it difficult to extract useful information. According to the characteristics of the temporary anchoring UUV in the polar region, the initial UUV alignment corresponds to the rocking base type. Polar coarse alignment process and polar fine alignment process constitute a complete polar initial alignment process [8,9].

The coarse alignment process focuses on the requirements of the alignment speed. The initial attitude matrix must be determined as soon as possible and the initial misalignment angle is limited within a certain range. Many coarse alignment algorithms have been proposed for the non-polar region such as analytic coarse alignment algorithm, horizontal second-order leveling + azimuth estimation coarse alignment algorithm, coarse alignment algorithm based on attitude matrix in inertial reference system, coarse alignment algorithm based on quaternions in inertial reference system, and so on [10]. In the analytic coarse alignment, the initial attitude matrix is obtained from the projection of the gravitational acceleration and the angular velocity of earth rotation in two frames. This simple coarse alignment algorithm can meet the general requirements of coarse alignment. The first step of horizontal second-order leveling + azimuth estimation coarse alignment is horizontal second-order leveling. After this, the control information is used as a reference correction to estimate the azimuth. This algorithm is widely used in engineering. However, these two coarse alignment algorithms are only suitable for the stationary base case. The coarse alignment algorithm based on attitude matrix in inertial reference system utilizes the characteristics that the direction can be extracted from the direction change of the gravitational acceleration in the inertial space. The coarse alignment algorithm based on quaternions in inertial reference system is nearly the same with the coarse alignment algorithm based on attitude matrix. Since both the quaternions and the attitude matrix are the representations of attitude and they can be transformed into each other. They are suitable for the rocking base case. However, they can only be used in the non-polar region. In the polar region, the Earth meridians converge fast, and the conventional initial alignment errors increase rapidly or even overflow.

After the coarse alignment, the fine alignment is used to improve the accuracy of the initial alignment [11]. The attitude error obtained after coarse alignment is relatively large. The deviation angle between the calculation navigation frame and the ideal navigation frame is the misalignment angle. In the non-polar region, a lot of filtering methods have been used to estimate the misalignment angle, such as Kalman filter (KF), Extend Kalman filter (EKF), Cubature Kalman filter (CKF), Unscented particle filter (UPF) and so on [12,13,14,15]. The estimated misalignment angle is used to correct the initial alignment results. The accuracy and the complexity of the filtering methods are different from each other. Like the conventional coarse alignment algorithm, the conventional fine alignment algorithm also has difficulties when used in the polar region. In the polar region, the fast convergence of the Earth meridians causes difficulties in the application of the conventional SINS. Small measurement errors would lead to significant calculation errors when a conventional SINS helps a UUV sailing in the polar region. Errors of the instruction angular velocity increase rapidly or even overflow in high latitude areas [16], making both the conventional SINS and conventional initial alignment algorithm invalid. A polar grid navigation algorithm is introduced to solve this problem. 

A polar initial alignment algorithm for polar UUV navigation is proposed in this paper. The main contribution of this paper is solving the problems that make the conventional initial alignment invalid in the polar region. Based on the characteristics of the polar region and UUV, polar initial alignment algorithm is designed for polar UUV navigation. The remaining sections are arranged as follows: the grid frame and the matrix representation are introduced in Section 2; Section 3 and Section 4 describe the polar coarse alignment algorithm and polar fine alignment algorithm for UUV, respectively; Simulation and experiment results are given in Section 5. Analyses and the invalid aspects of the conventional initial alignment algorithm are discussed in Section 6; Finally, the conclusions are summarized in Section 7.

## 2. Propaedeutic Conception

The Earth meridians converge rapidly in high latitude areas. That causes the calculation overflow and inaccuracy in conventional algorithms. Considering the invalid aspects of conventional SINS in the polar region, our polar grid navigation algorithm and the grid frame are introduced as follows. After the polar coarse alignment, the fine alignment is established based on the small misalignment angle assumption. The matrix representation of the frames with small rotation angle is useful to establish the kinematics model of UUV in the polar region. This matrix representation is discussed as follows.

### 2.1. The Frames

**Figure 1:**
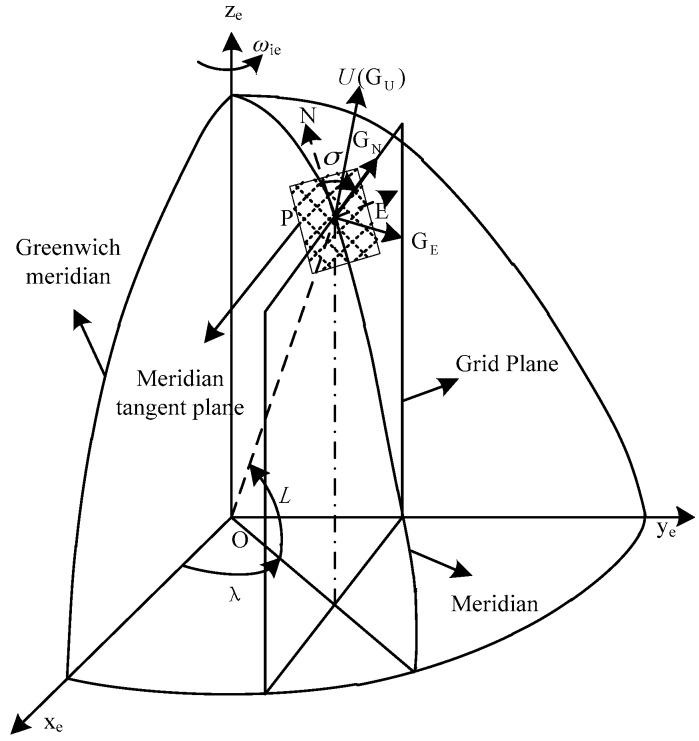
The description of the grid frame.

The frames are very important to the attitude description. The relationships among the frames reflect the attitude of UUV. Several frames are involved in this paper. They are the *i*, *e*, *b*, *g*, *G*, *n*, *b*_0_ and *o* frames. The *i* frame represents the inertial frame; *e* frame represents the Earth-centered Earth fixed frame; *b* frame represents the body frame of the UUV; *g* frame represents the geography frame; *G* frame represents the grid frame [17]; *n* frame represents the navigation frame; *b*_0_ frame represents the initial body frame solidified to the inertial frame and *o* frame represents the body frame of OCTANS. These frames are common except the *G* frame and *b*_0_ frame. The grid frame will be discussed in detail as follows:

As shown in Figure 1, point *O* and point *P* represent the center of the Earth and the location of the UUV, respectively. The latitude and longitude of point *P* are defined as *L* and *λ*, respectively. In order to describe the formation of the *G* frame, the following definitions are given first:

Grid north direction (*PG_N_*)—the intersecting line of the grid plane and local-level plane;
Grid up direction (*PG_U_*)—the direction coinciding with the geographic up axis;
Grid east direction (*PG_E_*)—the axis that forms a right-handed frame with the grid north axis and grid up axis.

Thus, *PG_E_G_N_G_U_* means the grid frame (*G* frame). There is an angle *σ* between the geographic north and grid north directions. This angle can be calculated from:

(1) $$\sin\sigma =\sin\lambda \sin L/\sqrt{1-\cos^{2}L\sin^{2}\lambda },$$

(2) $$\;\cos\sigma =\cos\lambda /\sqrt{1-\cos^{2}L\sin^{2}\lambda }.$$

The direction cosine matrix among the *e* frame, *g* frame and *G* frame are an orthogonal matrix. They can be expressed as:

(3) $$C_{e}^{g}=\left[\begin{array}{ccc} -\sin\lambda & \cos\lambda & 0\\ -\sin L\cos\lambda & -\sin L\sin\lambda & \cos L\\ \cos L\cos\lambda & \cos L\sin\lambda & \sin L \end{array}\right],$$

(4) $$C_{g}^{G}=\left[\begin{array}{ccc} \cos\sigma & -\sin\sigma & 0\\ \sin\sigma & \cos\sigma & 0\\ 0 & 0 & 1 \end{array}\right],$$

(5) $$C_{g}^{e}={\left(C_{e}^{g}\right)}^{-1}={\left(C_{e}^{g}\right)}^{T}=\left[\begin{array}{ccc} -\sin\lambda & -\sin L\cos\lambda & \cos L\cos\lambda \\ \cos\lambda & -\sin L\sin\lambda & \cos L\sin\lambda \\ 0 & \cos L & \sin L \end{array}\right].$$

### 2.2. The Matrix Representation of the Frames with Small Rotation Angle

Assuming that there is a small misalignment angle between the *Oxy*z coordinate system and the *Ox’y’z’* coordinate system, *Ox’y’z’* can be obtained from *Oxyz* though three small-angle rotations. The relation between *Oxyz* and *Ox’y’z’* can be described as:

(6) $$Oxyz\overset{around\;x\;axis}{\underset{\varphi_{x}}{\to }}Ox_{1}y_{1}z_{1}\overset{around\;y\;axis}{\underset{\varphi_{y}}{\to }}Ox_{2}y_{2}z_{2}\overset{around\;z\;axis}{\underset{\varphi_{z}}{\to }}Ox^{\prime }y^{\prime }z^{\prime }.$$

Considering that $\varphi_{x}$, $\varphi_{y}$ and $\varphi_{z}$ are small, then sin φ and cos φ can be approximately regarded as φ and 1, respectively. These small angles can be expressed in vector form as φ = [$\varphi_{x}$, $\varphi_{y}$, $\varphi_{z}$]*T*. Neglecting the second-order small terms, the relationship between *Oxy*z and *Ox’y’z’* in form of matrix can be written as:

(7) $$\left[\begin{array}{l} x^{\prime }\\ y^{\prime }\\ z^{\prime } \end{array}\right]=(I-\varphi \times )\left[\begin{array}{l} x\\ y\\ z \end{array}\right],$$

where, φ× is the anti-symmetric matrix of φ and it can be defined as:

(8) $$\varphi \times =\left[\begin{array}{ccc} 0 & -\varphi_{z} & \varphi_{y}\\ \varphi_{z} & 0 & -\varphi_{x}\\ -\varphi_{y} & \varphi_{x} & 0 \end{array}\right].$$

## 3. Polar Coarse Alignment Algorithm for UUV

Essentially, the purpose of the polar coarse alignment algorithm is to determine the rough initial attitude matrix of an UUV in the polar region. The gravitational acceleration follows a conical slow drift process in the inertial coordinate system. Thus, the output of the gyroscope can be used to track the motion of the angle in the UUV. The coarse alignment can be realized by the principle of the conical slow drift. How to extract the gravitational acceleration from the output of IMU is the key to realizing the coarse alignment. In the rocking base, the frequency of the gravitational acceleration is lower than that of the interference acceleration, therefore, a low pass filter can extract the gravitational acceleration or the interference acceleration can be eliminated by the integral smoothing method.

In the coarse alignment process of UUV, we focus on the alignment speed. The OCTANS is composed of three fiber-optic gyroscopes and three quartz accelerometers. It can only provide the attitude information of UUV quickly and accurately. However, the SINS consists of the IMU, the navigation solution unit and electronic equipment cabinet, etc. The attitude, velocity and position information of UUV all can be provided by SINS. SINS can be used for UUV initial alignment and navigation alone. The results of coarse alignment based on the SINS in UUV can meet the requirement of coarse alignment accuracy. Therefore, in the coarse alignment process of UUV, only SINS in UUV is considered. While in the fine alignment process of UUV, OCTANS is used for the assistant to improve the alignment accuracy.

Considering that conventional SINS is invalid in the polar region, polar grid navigation algorithm and the grid frame are introduced. In polar coarse alignment algorithm, G frame is chosen as n frame. The initial attitude matrix of UUV in the polar region can be obtained as:

(9) $$C_{b}^{G}=C_{e}^{G}C_{i}^{e}C_{b_{0}}^{i}C_{b}^{b_{0}},$$

where $C_{b}^{G}$ is the direction cosine matrix from *b* frame to *G* frame and it is the attitude matrix of UUV; $C_{e}^{G}$ is the direction cosine matrix from *e* frame to *G* and it is determined by the location of UUV; $C_{i}^{e}$ is the direction cosine matrix from *i* frame to *e* frame and it is related to the time interval Δt; $C_{b_{0}}^{i}$ is the direction cosine matrix from *b*_0_ frame to *i* frame; $C_{b}^{b_{0}}$ is the direction cosine matrix from *b* frame to *b*_0_ frame and $C_{b}^{b_{0}}(t_{0})=I$ at the initial time *t*_0_. The following $C_{b}^{b_{0}}(t)$ can be updated based on the output of the gyroscope:

(10) $$C_{e}^{G}=C_{e}^{g}C_{g}^{G}=\left[\begin{array}{ccc} -c\sigma s\lambda \;+\;s\sigma c\lambda sL & c\sigma c\lambda \;+\;s\sigma sLs\lambda & -s\sigma cL\\ -s\sigma s\lambda -c\sigma sLc\lambda & s\sigma c\lambda -c\sigma sLs\lambda & c\sigma cL\\ cLc\lambda & cLs\lambda & sL \end{array}\right],$$

where c(⋅) and s(⋅) represent cos(⋅) and sin(⋅), respectively.

(11) $$C_{i}^{e}=\left[\begin{array}{ccc} \cos\omega_{ie}\Delta t & \sin\omega_{ie}\Delta t & 0\\ -\sin\omega_{ie}\Delta t & \cos\omega_{ie}\Delta t & 0\\ 0 & 0 & 1 \end{array}\right],$$

where, $\omega_{ie}$ is the angular velocity of the earth rotation and $\Delta t=t-t_{0}$ is the time interval, $t_{0}$ is the initial time.

(12) $${\dot{C}}_{b}^{b_{0}}=C_{b}^{b_{0}}\left[\omega_{b_{0}b}^{b}\times \right]=C_{b}^{b_{0}}\left[\omega_{ib}^{b}\times \right],$$

where, $\left[\omega_{b_{0}b}^{b}\times \right]$ and $\left[\omega_{ib}^{b}\times \right]$ are the anti-symmetric matrix of $\omega_{b_{0}b}^{b}$ and $\omega_{ib}^{b}$, respectively. Since $b_{0}$ frame represents the initial body frame solidified to the inertial frame, $\omega_{b_{0}b}^{b}$ can be expressed as $\omega_{b_{0}b}^{b}=\omega_{ib}^{b}$. And $\omega_{ib}^{b}$ is the output of the gyroscope.

From the above analysis, $C_{e}^{G}$, $C_{i}^{e}$ and $C_{b}^{b_{0}}$ can be directly calculated based on the location of UUV and the outputs of IMU. In order to obtain the rough initial attitude matrix of UUV ($C_{b}^{G}$), the direction cosine matrix $C_{b_{0}}^{i}$ should be determined first. The conical slow drift process in the inertial coordinate system of the gravitational acceleration is used to determine $C_{b_{0}}^{i}$.

In *g* frame, the gravitational acceleration can be expressed as:

(13) $$g^{g}={\left[0\;0\;-g\right]}^{T}.$$

Based on the relationships among *i* frame, *e* frame and *g* frame, the gravitational acceleration in *i* frame can be described as:

(14) $$g^{i}=C_{e}^{i}C_{g}^{e}g^{g}={\left[\begin{array}{c} -g\cos L\cos(\lambda +\omega_{ie}\Delta t)\\ -g\cos L\sin(\lambda +\omega_{ie}\Delta t)\\ -g\sin L \end{array}\right]}^{T}.$$

To eliminate the interference acceleration, the integral smoothing method is introduced:

(15) $$\;V^{i}(t_{k})=\int_{t_{0}}^{t_{k}}-g^{i}dt.$$

By substituting Equation (14), Equation (15) can be rewritten as:

(16) $$V^{i}(t_{k})=\left[\begin{array}{c} \left(g\cos L\left[\sin\;(\lambda +\omega_{ie}\Delta t_{k})-\sin\lambda \right]\right)/\omega_{ie}\\ \left(g\cos L\left[\cos\lambda -\cos\;(\lambda +\omega_{ie}\Delta t_{k})\right]\right)/\omega_{ie}\\ g\sin L\Delta t_{k} \end{array}\right].$$

The output of the accelerometer $f^{b}$ is composed of gravitational acceleration $g^{b}$, the interference acceleration $a_{d}^{b}$ and accelerometer bias $\nabla^{b}$:

(17) $$\;f^{b}=-g^{b}+a_{d}^{b}+\nabla^{b}.$$

By substituting Equation (17), $f^{b_{0}}$ can be written as:

(18) $$\;f^{b_{0}}=C_{b}^{b_{0}}\left(-g^{b}+a_{d}^{b}+\nabla^{b}\right).$$

Using $V^{b_{0}}$ to represent the result of $f^{b_{0}}$ after the integral smoothing, it can be defined as:

(19) $$\begin{array}{ll} \;V^{b_{0}}\left(t_{k}\right) & =\int_{t_{0}}^{t_{k}}f^{b_{0}}dt=\int_{t_{0}}^{t_{k}}C_{b}^{b_{0}}\left(-g^{b}+a_{d}^{b}+\nabla^{b}\right)dt=\int_{t_{0}}^{t_{k}}-C_{b}^{b_{0}}g^{b}dt+\int_{t_{0}}^{t_{k}}C_{b}^{b_{0}}\left(a_{d}^{b}+\nabla^{b}\right)dt\\ & =C_{i}^{b_{0}}\int_{t_{0}}^{t_{k}}-g^{i}dt+\int_{t_{0}}^{t_{k}}C_{b}^{b_{0}}\left(a_{d}^{b}+\nabla^{b}\right)dt \end{array}.$$

Considering $a_{d}^{b}$ and $\nabla^{b}$ are little and the frequency of $a_{d}^{b}$ is high, the assumptions can be made as:

(20) $$\;\int_{t_{0}}^{t_{k}}C_{b}^{b_{0}}\left(a_{d}^{b}+\nabla^{b}\right)dt=0.$$

By substituting Equation (20), Equation (19) can be rewritten as:

(21) $$\;V^{b_{0}}\left(t_{k}\right)=C_{i}^{b_{0}}\int_{t_{0}}^{t_{k}}-g^{i}dt=C_{i}^{b_{0}}V^{i}\left(t_{k}\right),$$

And at the moment $t_{k1}$ and $t_{k2}$($\;t_{0}<t_{k1}<t_{k2}$), $V^{b_{0}}\left(t_{k}\right)$ can be obtained as:

(22) $$V^{b_{0}}\left(t_{k1}\right)=C_{i}^{b_{0}}V^{i}\left(t_{k1}\right),$$

(23) $$\;V^{b_{0}}\left(t_{k2}\right)=C_{i}^{b_{0}}V^{i}\left(t_{k2}\right),$$

(24) $$\;V^{b_{0}}\left(t_{k1}\right)\times V^{b_{0}}\left(t_{k2}\right)=\left[C_{i}^{b_{0}}V^{i}\left(t_{k1}\right)\right]\times \left[C_{i}^{b_{0}}V^{i}\left(t_{k2}\right)\right]=C_{i}^{b_{0}}\left[V^{i}\left(t_{k1}\right)\times V^{i}\left(t_{k2}\right)\right].$$

Based on Equations (22)–(24), $C_{b_{0}}^{i}$ can be calculated as:

(25) $$\;C_{b_{0}}^{i}=\left[\begin{array}{c} {\left[V^{i}\left(t_{k1}\right)\right]}^{T}\\ {\left[V^{i}\left(t_{k2}\right)\right]}^{T}\\ {\left[V^{i}\left(t_{k1}\right)\times V^{i}\left(t_{k2}\right)\right]}^{T} \end{array}\right]{\left[\begin{array}{c} {\left[V^{b_{0}}\left(t_{k1}\right)\right]}^{T}\\ {\left[V^{b_{0}}\left(t_{k2}\right)\right]}^{T}\\ {\left[V^{b_{0}}\left(t_{k1}\right)\times V^{b_{0}}\left(t_{k2}\right)\right]}^{T} \end{array}\right]}^{-1}.$$

By substituting Equations (10)–(12) and (25) into Equation (9), the rough initial attitude matrix of the UUV in the polar region can be determined and the polar coarse alignment is accomplished.

## 4. Polar Fine Alignment Algorithm for UUV

The main purpose of the fine alignment is to estimate and compensate the misalignment angle based on the filter. The misalignment angle is applied to the correction of the initial alignment system. Polar fine alignment is based on the results of the polar coarse alignment mentioned in Section 3. Therefore, the misalignment angle is satisfied with the assumption of small misalignment angle. Like the conventional coarse alignment algorithm, the conventional fine alignment algorithm has difficulties for using it in the polar region. To solve the problem of invalid conventional fine alignment algorithm, polar grid navigation algorithm and the grid frame are also introduced in this section. The grid frame is chosen as the navigation frame in polar fine alignment algorithm. Based on the assumption of temporary anchoring UUV, the rocking base is considered in this paper. According the characteristics of UUV, the misalignment angle and the velocity error are chosen as the observations. A Kalman filter (KF) is used to estimate the misalignment angle and the velocity error. This filter algorithm can meet the requirement of alignment accuracy. And KF is uncomplicated compared with other filter algorithms.

### 4.1. Attitude Error Equation

After the polar coarse alignment, the misalignment angle $\phi^{G}={\left[\phi_{x}^{G}\;\phi_{y}^{G}\;\phi_{z}^{G}\right]}^{T}$ is small. $\phi^{G}={\left[\phi_{x}^{G}\;\phi_{y}^{G}\;\phi_{z}^{G}\right]}^{T}$ represents the misalignment angle between ideal *G* frame and actual *G* frame (*G’* frame). The attitude update equations of UUV in ideal condition and in actual condition can be described as follows, respectively:

(26) $${\dot{C}}_{b}^{G}=C_{b}^{G}\left[\omega_{ib}^{b}\times \right]-\left[\omega_{iG}^{G}\times \right]C_{b}^{G},$$

(27) $$\;{\dot{C}}_{b}^{G^{\prime }}=C_{b}^{G^{\prime }}\left[\left(\omega_{ib}^{b}+\delta \omega_{ib}^{b}\right)\times \right]-\left[\left(\omega_{ie}^{G}+\delta \omega_{ie}^{G}+\omega_{eG}^{G}+\delta \omega_{eG}^{G}\right)\times \right]C_{b}^{G^{\prime }},$$

where Equation (26) is the attitude update equation of UUV in ideal condition and Equation (27) is the attitude update equation of UUV in actual condition. Considering the errors in actual condition, $\delta \omega_{ib}^{b}$, $\delta \omega_{ie}^{G}$ and $\delta \omega_{eG}^{G}$ represent the errors of $\omega_{ib}^{b}$, $\omega_{ie}^{G}$ and $\omega_{eG}^{G}$, respectively.

(28) $$\;\delta \omega_{ib}^{b}=\varepsilon^{b}=\varepsilon_{c}^{b}+\varepsilon_{w}^{b},$$

where $\varepsilon^{b}$ is the gyro drifts; $\varepsilon_{c}^{b}$ is the gyro constant drifts and $\varepsilon_{w}^{b}$ is the gyro random drifts. The gyro random drifts are assumed as white noise.

Considering Equations (26) and (27), the attitude error equation of UUV can be obtained as:

(29) $$\;{\dot{\phi }}^{G}=-(\omega_{iG}^{G}\times )\phi^{G}+C_{\omega eGv}\delta V^{G}-C_{b}^{G^{\prime }}\varepsilon^{b},$$

where $\delta V^{G}={\left[\delta v_{GE}\;\delta v_{GN}\;\delta v_{GU}\right]}^{T}$ is the velocity error of UUV and $C_{\omega eGv}$ can be expressed as:

(30) $$\;C_{\omega eGv}=\left[\begin{array}{ccc} 0 & -1/R_{eh} & 0\\ 1/R_{eh} & 0 & 0\\ 0 & -\left(\cot L\sin\sigma \right)/R_{eh} & 0 \end{array}\right],$$

where $R_{eh}$ is the radius of the Earth.

### 4.2. Velocity Error Equation

The velocity differential equation of the UUV in ideal conditions and in actual conditions can be described as follows, respectively:

(31) $${\dot{V}}^{G}=C_{b}^{G}f^{b}-\left(2\omega_{ie}^{G}+\omega_{eG}^{G}\right)\times V^{G}+g^{G},$$

(32)  V^˙G=(I−ϕG×)CbG(fb+∇b)+(gG+δgG)−[2(ωieG+δωieG)+(ωeGG+δωeGG)] ×(VG+δVG),

where Equation (31) is the velocity differential equation of UUV in ideal condition and Equation (32) is the velocity differential equation of UUV in actual condition. The errors of $V^{G}$, $f^{b}$, $\omega_{ie}^{G}$, $\omega_{eG}^{G}$ and $g^{G}$ in actual condition are described as $\delta V^{G}$, $\nabla^{b}$, $\delta \omega_{ie}^{G}$, $\delta \omega_{eG}^{G}$ and $\delta g^{G}$, respectively:

(33) $$\;\nabla^{b}=\nabla_{c}^{b}+\;\nabla_{w}^{b},$$

where $\nabla^{b}$ is the accelerometer bias; $\nabla_{c}^{b}$ is the accelerometer constant bias and $\nabla_{w}^{b}$ is the accelerometer random bias. The accelerometer random bias is assumed as white noise.

Combing Equations (31) and (32), the velocity error equation of UUV can be described as:

(34) $$\;\delta {\dot{V}}^{G}=f^{G}\times \phi^{G}+C_{b}^{G}\nabla^{b}\;+[V^{G}\times C_{\omega eGv}-\left(2\omega_{ie}^{G}+\omega_{eG}^{G}\right)\times ]\;\cdot \delta V^{G},$$

### 4.3. Error Model of OCTANS

There are attitude errors in SINS. To obtain the accuracy results, these attitude errors are corrected by OCTANS. OCTANS is an all-in-one gyrocompass and motion sensor produced by iXblue (Paris, France). It is composed of three fiber-optic gyroscopes and three quartz accelerometers. The precision of OCTANS is within 0.01°. Therefore, OCTANS realizes the attitude correction during the polar fine alignment. The error model of OCTANS can be built as:

(35) $$\;\varepsilon_{o}^{o}=\varepsilon_{wo}^{o}+\varepsilon_{co}^{o},$$

where $\varepsilon_{o}^{o}$ is the gyro drifts composed of the gyro constant drifts $\varepsilon_{co}^{o}$ and gyro random drifts $\varepsilon_{wo}^{o}$. And the gyro constant drifts can be expressed as:

(36) $$\;{\dot{\varepsilon }}_{co}^{o}=0.$$

Neglecting the installation error angles, the projection of Equation (35) in *G* frame can be described as:

(37) $$\;\varepsilon_{o}^{G}=C_{b}^{G}C_{o}^{b}\varepsilon_{o}^{o}=C_{b}^{G}I\left(\varepsilon_{wo}^{o}+\varepsilon_{co}^{o}\right)=\varepsilon_{wo}^{G}+\varepsilon_{co}^{G}.$$

### 4.4. Dynamic Model

The states consist of the attitude error of SINS, $\phi^{G}$, the velocity error of SINS, $\delta V^{G}$, the gyro constant drifts and the accelerometer bias in SINS, $\varepsilon_{c}^{b}$ and $\nabla_{c}^{b}$, and the gyro constant drifts of OCTANS, $\varepsilon_{co}^{o}$. These states can be described as:

(38) $$\;X={\left[{\left(\phi^{G}\right)}^{T}\;{\left(\delta V^{G}\right)}^{T}\;{\left(\varepsilon_{c}^{b}\right)}^{T}\;{\left(\nabla_{c}^{b}\right)}^{T}\;{\left(\varepsilon_{co}^{o}\right)}^{T}\right]}^{T}.$$

Based on the states and the error equations including Equations (29), (34) and (36), the dynamic model of the UUV can be defined as:

(39) $$\;\left\{ \begin{array}{l} {\dot{\phi }}^{G}=-(\omega_{iG}^{G}\times )\phi^{G}+C_{\omega eGv}\delta V^{G}-C_{b}^{G^{\prime }}\varepsilon^{b}\\ \delta {\dot{V}}^{G}=f^{G}\times \phi^{G}+C_{b}^{G}\nabla^{b}\\ \;+[V^{G}\times C_{\omega eGv}-\left(2\omega_{ie}^{G}+\omega_{eG}^{G}\right)\times ]\;\cdot \delta V^{G}\\ {\dot{\varepsilon }}^{b}\;=\;0\\ {\dot{\nabla }}^{b}\;=\;0\\ {\dot{\varepsilon }}_{co}^{o}=0 \end{array}\right..$$

Equation (39) is rewritten in form of vector as:

(40) $$\;\dot{X}=AX+BW.$$

Making comparison between Equations (39) and (40), the system matrix, A, the control matrix, B, and the system noise matrix, W, can be obtained as follows:

$A=\left[\begin{array}{ccccc} A_{1} & A_{2} & 0_{3\times 3} & A_{3} & 0_{3\times 3}\\ A_{4} & A_{5} & 0_{3\times 3} & 0_{3\times 3} & A_{6}\\ 0_{3\times 3} & 0_{3\times 3} & 0_{3\times 3} & 0_{3\times 3} & 0_{3\times 3}\\ 0_{3\times 3} & 0_{3\times 3} & 0_{3\times 3} & 0_{3\times 3} & 0_{3\times 3}\\ 0_{3\times 3} & 0_{3\times 3} & 0_{3\times 3} & 0_{3\times 3} & 0_{3\times 3} \end{array}\right]，$ $B=\left[\begin{array}{cc} B_{1} & 0_{3\times 3}\\ 0_{3\times 3} & B_{2}\\ 0_{3\times 3} & 0_{3\times 3}\\ 0_{3\times 3} & 0_{3\times 3}\\ 0_{3\times 3} & 0_{3\times 3} \end{array}\right]，$ $W={\left[{\left(\varepsilon_{r}^{b}\right)}^{T}\;{\left(\nabla_{r}^{G}\right)}^{T}\right]}^{T}$ where $A_{1}=-(\omega_{iG}^{G}\times )$, $A_{2}=C_{\omega eGv}$, $A_{3}=-C_{b}^{G^{\prime }}$, $A_{4}=\left(f^{G}\times \right)$, $A_{5}=\left[V^{G}\times C_{\omega eGv}-\left(2\omega_{ie}^{G}+\omega_{eG}^{G}\right)\times \right]$, $A_{6}=C_{b}^{G}$, $B_{1}=-C_{b}^{G^{\prime }}$, $B_{2}=C_{b}^{G}$.

### 4.5. Observation Model

The output of high-precision OCTANS is chosen as the attitude reference. The polar fine alignment algorithm for UUV is based on the rocking base. The initial velocity is regarded as zero. The attitude errors and the velocity errors are chosen as the observation states:

(41) $$\;Z={\left[\begin{array}{cc} {\left(\phi^{G}\right)}^{T} & {\left(\delta V^{G}\right)}^{T} \end{array}\right]}^{T}.$$

The gyro constant drifts of OCTANS are assumed to be modeled well and estimated well. This gyro constant can be compensated to the output of OCTANS. The projection of the OCTANS outputs after partial error compensation in the grid frame can be written as:

(42) $$\;{\hat{\Lambda }}_{o}^{G}=\Lambda_{o}^{G}+\varepsilon_{wo}^{G}={\tilde{\Lambda }}_{o}^{G}-\varepsilon_{co}^{G},$$

where the attitude of UUV is $\Lambda ={\left[\theta \;\psi \right]}^{T}$ including roll angle, pitch angle and yaw angle. ${\hat{\Lambda }}_{o}^{G}$ is the attitude measured by OCTANS after partial error compensation; $\Lambda_{o}^{G}$ is the ideal attitude; ${\tilde{\Lambda }}_{o}^{G}$ is the actual attitude measured by OCTANS and $\varepsilon_{wo}^{G}$ is the zero-mean Gaussian white noise.

The observation model can be built as:

(43) $$\;Z=\left[\begin{array}{c} {\hat{\Lambda }}_{o}^{G}-\Lambda^{G}\\ V^{G} \end{array}\right]=\left[\begin{array}{c} \phi^{G}+\varepsilon_{wo}^{G}\\ \delta V^{G}+v^{G} \end{array}\right],$$

where $\Lambda^{G}$ is the attitude output of SINS and $V^{G}$ is the velocity output of SINS.

Equation (42) is rewritten in form of vector as:

(44) $$\;\dot{Z}=HX+V,$$

where the measurement matrix is:

$$H={\left[\begin{array}{lllll} I_{3\times 3} & 0_{3\times 3} & 0_{3\times 3} & 0_{3\times 3} & 0_{3\times 3}\\ 0_{3\times 3} & I_{3\times 3} & 0_{3\times 3} & 0_{3\times 3} & 0_{3\times 3} \end{array}\right]}^{T}$$

and the measurement noise is $V={\left[{\left(\varepsilon_{wo}^{G}\right)}^{T}\;{\left(v^{G}\right)}^{T}\right]}^{T}$ which is regarded as the white noise and V∼N(0,R); R is the measurement noise covariance.

## 5. Simulation and Experiment Results

Based on the assumption of the temporary anchoring UUV, the polar initial alignment algorithm proposed in this paper is based on the rocking base. The polar initial alignment algorithm proposed in this paper is compared with conventional and transversal initial alignment algorithms for polar UUV navigation. In coarse alignment, the coarse alignment model proposed in paper [18] is chosen as the comparison model. It is called conventional coarse alignment. In fine alignment, the fine alignment model proposed in paper [19] is chosen as the comparison model. It is called conventional fine alignment. The initial alignment algorithm based on transversal navigation algorithm [20] is also chosen as the comparison model. It is called transversal coarse alignment algorithm and transversal fine alignment algorithm. Simulation and experiment are conducted to verify the availability of the proposed polar initial alignment algorithm for polar UUV navigation.

### 5.1. Simulation Results and Analyses

The polar initial alignment algorithm for polar UUV navigation consists of polar coarse and fine alignment algorithms. The polar coarse alignment algorithm and polar fine alignment algorithm are verified by simulation in this section, respectively. To satisfy with alignment accuracy and alignment speed, the simulation time of polar coarse alignment algorithm is set as 120 s and the simulation time of polar fine alignment algorithm is set as 1200 s. The results of the polar coarse alignment, the mean of the attitude errors, are set as the initial states of polar fine alignment. Other relevant parameters are the same for both polar coarse and fine alignment algorithms. For the filter in polar fine alignment algorithm, the initial state estimation covariance matrix $P_{0}$, system noise covariance Q, and measurement noise covariance matrix R are set as follows:

$$P_{0}=diag\left\{\begin{array}{l} {\left(0.01\pi /180\;\mathrm{rad}\right)}^{2},{\left(0.01\pi /180\;\mathrm{rad}\right)}^{2},{\left(0.01\pi /180\;\mathrm{rad}\right)}^{2},\\ {\left(0.1\;m/s\right)}^{2},{\left(0.1\;m/s\right)}^{2},{\left(0.1\;m/s\right)}^{2},{\left(0.03\pi /180/3600\;\mathrm{rad}/s\right)}^{2},\\ {(0.03\pi /180/3600\;\mathrm{rad}/s)}^{2},{\left(0.03\pi /180/3600\;\mathrm{rad}/s\right)}^{2}，\\ {\left(1\times 10^{-4}g_{0}\;m/s^{2}\right)}^{2},{\left(1\times 10^{-4}g_{0}\;m/s^{2}\right)}^{2},{\left(1\times 10^{-4}g_{0}\;m/s^{2}\right)}^{2},\\ {\left(0.01\pi /180/3600\;\mathrm{rad}/s\right)}^{2},{\left(0.01\pi /180/3600\;\mathrm{rad}/s\right)}^{2},\\ {\left(0.01\pi /180/3600\;\mathrm{rad}/s\right)}^{2} \end{array}\right\}$$

$$Q=diag\left\{\begin{array}{l} {\left(0.001\pi /180/3600\;\mathrm{rad}/s\right)}^{2},{\left(0.001\pi /180/3600\;\mathrm{rad}/s\right)}^{2},\\ {\left(0.001\pi /180/3600\;\mathrm{rad}/s\right)}^{2},{\left(1\times 10^{-5}g_{0}\;m/s^{2}\right)}^{2},\\ {\left(1\times 10^{-5}g_{0}\;m/s^{2}\right)}^{2},{\left(1\times 10^{-5}g_{0}m/s^{2}\right)}^{2} \end{array}\right\}$$

$$R=diag\left\{\begin{array}{l} {\left(0.01\pi /180\;\mathrm{rad}\right)}^{2},{\left(0.01\pi /180\;\mathrm{rad}\right)}^{2},\\ {\left(0.01\pi /180\;\mathrm{rad}\right)}^{2},{\left(0.01\;m/s\right)}^{2},\\ {\left(0.01\;m/s\right)}^{2},{\left(0.01\;m/s\right)}^{2} \end{array}\right\}$$

The other parameters for simulation are set as Table 1. These parameters are simulation time (coarse/fine), filtering period, latitude, longitude, amplitude of pitch/roll/yaw, period of pitch/roll/yaw, gyro constant drifts, gyro random drifts, accelerometer constant bias and accelerometer random bias.

The coarse alignments are conducted 100 times based on polar, conventional and transversal coarse alignment algorithm for polar UUV navigation, respectively. 

**Figure 2:**
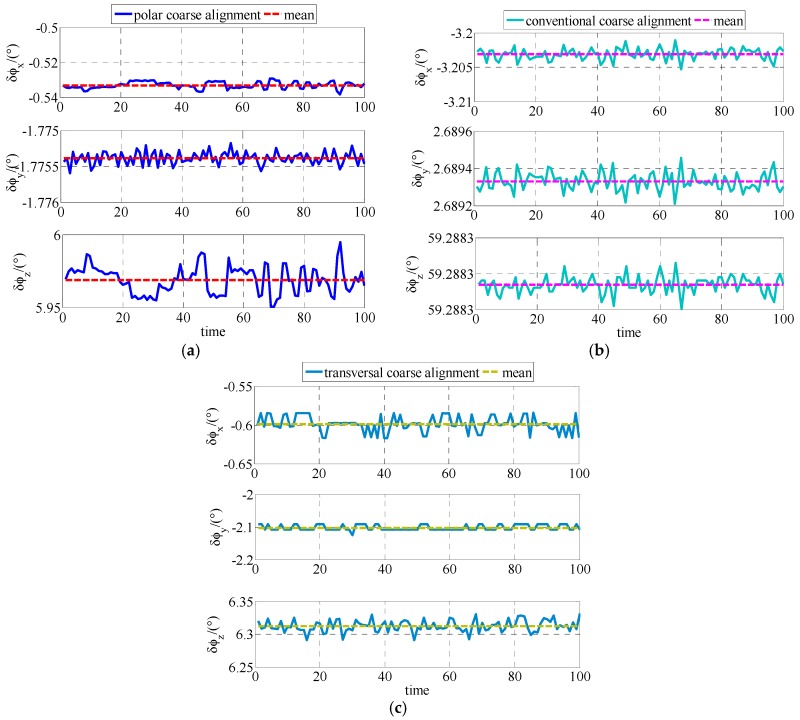
Estimates of $\phi^{G}$ in simulation: (**a**) Estimates of $\phi^{G}$ based on polar coarse alignment algorithm for polar UUV navigation; (**b**) Estimates of $\phi^{G}$ based on conventional coarse alignment algorithm for polar UUV navigation; (**c**) Estimates of $\phi^{G}$ based on transversal coarse alignment algorithm for polar UUV navigation.

The simulation results are expressed for comparison shown as Figure 2 and Table 2. As shown in Figure 2, the coarse alignment results of polar, conventional and transversal coarse alignment algorithms are all stable near a value, respectively. Statistical results about the comparison simulation between polar, conventional and transversal coarse alignment algorithms are shown as Table 2. 

Based on the results shown in Table 2, the following conclusions about the simulation results can be made. After polar coarse alignment, the mean value of east misalignment angle, north misalignment angle and up misalignment angle are 0.5331°, −1.7753° and 5.9691°, respectively. After conventional coarse alignment, the mean value of east misalignment angle, north misalignment angle and up misalignment angle are −3.2030°, 2.6893° and 59.2882°, respectively. After transversal coarse alignment , the mean value of east misalignment angle, north misalignment angle and up misalignment angle are −0.5982°, −2.1027° and 6.3123°, respectively. The standard deviation of polar coarse alignment, conventional coarse alignment and transversal coarse alignment are not more than 0.570818, 0.002867 and 0.602804, respectively. The polar coarse alignment algorithm is much superior to conventional coarse alignment algorithm and transversal coarse alignment for polar UUV navigation.

**Figure 3:**
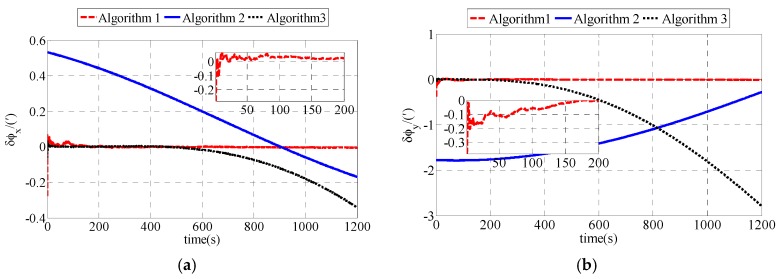

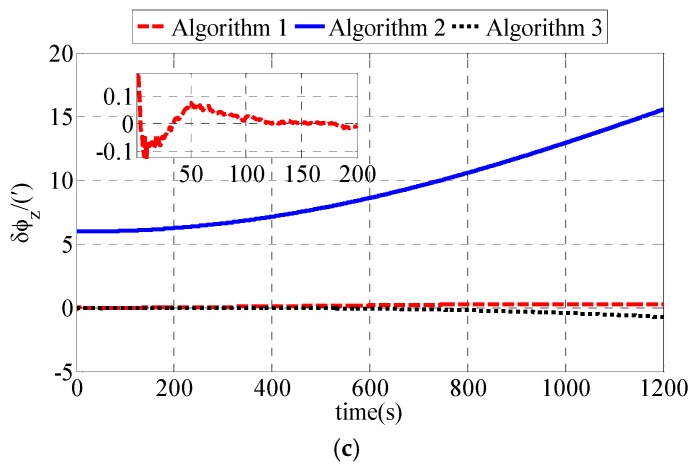
Estimation errors of $\phi^{G}$ in simulation: (**a**) Estimation errors of $\phi_{x}^{G}$; (**b**) Estimation errors of $\phi_{y}^{G}$; (**c**) Estimation errors of $\phi_{z}^{G}$.

The fine alignments are also compared among polar, conventional and transversal fine alignment algorithms. To simplify the expression, the polar fine alignment algorithm proposed in this paper is defined as Algorithm 1. The conventional fine alignment algorithm is defined as Algorithm 2. And the transversal fine alignment algorithm is defined as Algorithm 3. Simulation results are shown as Figure 3.

As shown in Figure 3, the estimation errors of polar fine alignment algorithm are less than those of conventional and transversal fine alignment algorithms. Compared with the estimation errors of conventional and transversal fine alignment algorithms, the estimation errors of polar fine alignment algorithm are closer to zero. The estimation errors of polar fine alignment algorithm for polar UUV navigation converge to near zero within 200 s. Statistical results of fine alignment algorithms are shown as Table 3.

As shown in Table 3, the estimation errors of *δφ_x_*, *δφ_y_* and *δφ_z_* in the polar fine alignment algorithm proposed in this paper are 0.0060′, 0.0039′ and 0.2143′, respectively. The estimation errors of *δφ_x_*, *δφ_y_* and *δφ_z_* in the conventional fine alignment algorithm for polar UUV navigation are 0.0481′, 0.0202′ and 14.8649′, respectively. The estimation errors of *δφ_x_*, *δφ_y_* and *δφ_z_* in transversal fine alignment algorithm for polar UUV navigation are 0.1303′, 1.2560′ and 0.2953′, respectively. The RMS errors of polar fine alignment algorithm proposed in this paper are much smaller than those of conventional fine alignment algorithm and transversal fine alignment algorithm for polar UUV navigation. The fine alignment results of our polar fine alignment algorithm are obviously superior to those of the conventional and transversal fine alignment algorithms for polar UUV navigation.

The computational time of polar coarse alignment algorithm and polar fine alignment algorithm proposed in this paper are 1.088663 s and 26.119527 s, respectively. The gyro drifts and the accelerometer bias of IMU are shown in Table 1. The accuracy of other hardware needed to support calculation is as follows. The gyro constant drifts and random drifts of OCTANS are 0.01 °/h and (0.0005 °/h)^2^, respectively [21,22]. Simulation results demonstrate that polar initial alignment algorithm proposed in this paper is obviously superior to conventional and transversal fine alignment algorithms in alignment accuracy and alignment speed.

### 5.2. Experiment Results and Analyses

Considering the restriction of geography, the experiment was conducted in form semi-physical simulation. The experimental data is composed of the practical measured data and the simulated data. The angular velocity ${\hat{\omega }}_{ib}^{b}$ and special force ${\hat{f}}^{b}$ compose the experimental data. The angular velocity ${\hat{\omega }}_{ib}^{b}$ consists of true angular velocity $\omega_{ib}^{b}$ and gyro drifts $\delta \omega_{ib}^{b}$. The special force ${\hat{f}}^{b}$ consists of true special force $f^{b}$ and accelerometer bias $\delta f^{b}$:

(45) $$\;{\hat{\omega }}_{ib}^{b}=\;\omega_{ib}^{b}+\;\delta \omega_{ib}^{b}=\;\omega_{ib}^{b}+\varepsilon^{b},$$

(46) $$\;{\hat{f}}^{b}=f^{b}+\;\delta f^{b}=f^{b}+\nabla^{b}.$$

**Figure 4:**
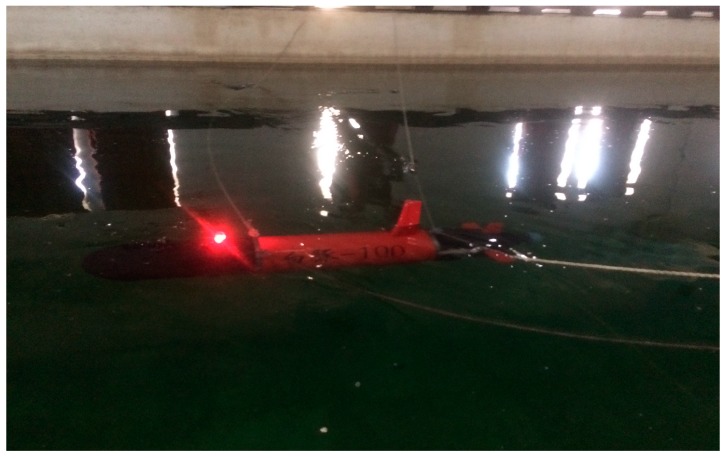
Unmanned Underwater Vehicle during the experiment.

No matter whether gained from simulation or experiment, the true values of IMU $\omega_{ib}^{b}$ and $f^{b}$ are the same. The values of $\omega_{ib}^{b}$, and $f^{b}$ can be gained from a simulation once the attitude variation and maneuvers of the UUV are confirmed [23]. Therefore, $\omega_{ib}^{b}$ and $f^{b}$ can be gained from simulation. The practical measured data consists of gyro drifts and the accelerometer bias that is supplied by the IMU in UUV. Since the gyro and accelerometer biases are the inherent characteristics of SINS in UUV, they would not change in different regions. Therefore, the gyro and accelerometer biases for the polar experiment can be measured in the non-polar region. The biases can be extracted from the practical measured data [24], which can be provided by the SINS in UUV shown in Figure 4. The simulated data includes the true values of the angular velocity and special force.

The practical measured data is gained from an experiment conducted in a rectangular pool located at the non-polar region (N45°73′ E127°41′). The UUV used for experiment is built by our laboratory and called White Dolphin-100. 

The sensors in the UUV include IMU, OCTANS, DVL, GPS, depth sensor, underwater camera and sonar. Among the sensors, IMU and OCTANS play important roles in the experiment. DVL and GPS provide initial velocity and position, respectively. The practical measured data extracted from the experiment can be expressed as Table 4. The conditions of the simulated data are the same with the relevant parameters in Section 5.1 except the following parameters:

$$Q_{e}=diag\left\{\begin{array}{l} {\left(4.102\times 10^{-6}\;\mathrm{rad}/s\right)}^{2},{\left(4.296\times 10^{-6}\;\mathrm{rad}/s\right)}^{2},{\left(2.375\times 10^{-6}\;\mathrm{rad}/s\right)}^{2},\\ {\left(0.00162\;m/s^{2}\right)}^{2},{\left(0.002001\;m/s^{2}\right)}^{2}\;,{\left(0.0007122\;m/s^{2}\right)}^{2} \end{array}\right\}$$

**Figure 5:**
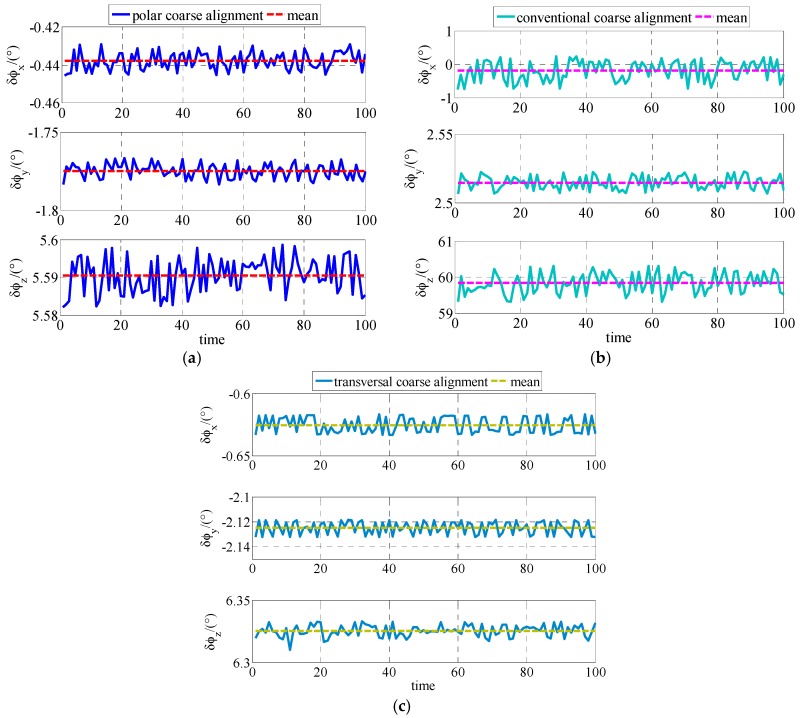
Estimates of $\phi^{G}$ in experiment: (**a**) Estimates of $\phi^{G}$ based on polar coarse alignment algorithm for polar UUV navigation; (**b**) Estimates of $\phi^{G}$ based on conventional coarse alignment algorithm for polar UUV navigation; (**c**) Estimates of $\phi^{G}$ based on transversal coarse alignment algorithm for polar UUV navigation.

The experiment is also conducted to be compared among the polar, conventional and transversal initial alignment algorithms for polar UUV navigation. The experiment results of coarse alignment comparison are shown as Figure 5 and Table 5. 

As shown in Figure 5, the misalignment angle of polar coarse alignment algorithm proposed in this paper is stable near a value as same as conventional and transversal coarse alignment algorithms for polar UUV navigation. The statistical results about polar, conventional and transversal coarse alignment algorithms for polar UUV navigation in experiment are expressed as Table 5.

As shown in Table 5, the misalignment angles of polar coarse alignment algorithm proposed in this paper are −0.4378°, −1.7750° and 5.5904°, respectively. In conventional coarse alignment algorithm for polar UUV navigation, the misalignment angles are −0.1959°, 2.5139° and 59.8336°, respectively. In transversal coarse alignment algorithm for polar UUV navigation, the misalignment angles are −0.6256°, −2.1249° and 6.3255°, respectively. The standard deviations of polar, conventional and transversal coarse alignment algorithms for polar UUV navigation are not more than 0.310097, 0.312622 and 0.385646, respectively. The polar coarse alignment algorithm proposed in this paper is obviously superior to the conventional and transversal coarse alignment algorithms for polar UUV navigation.

**Figure 6:**
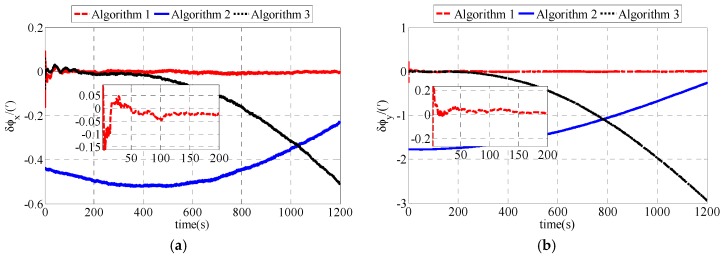

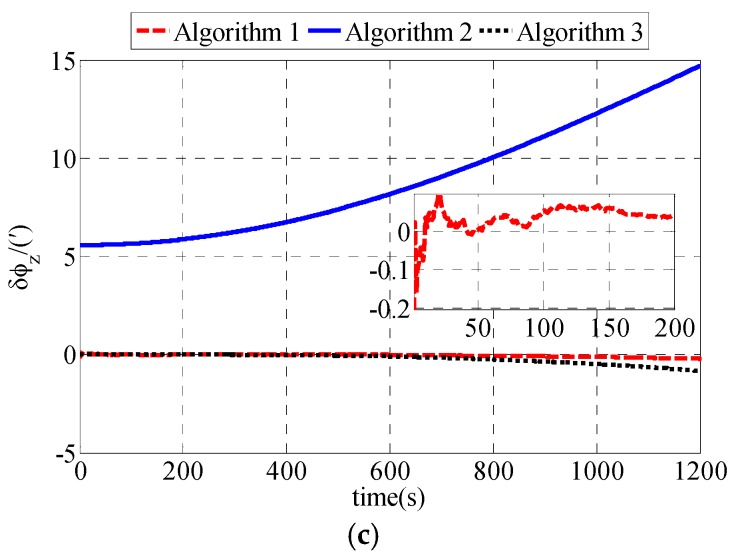
Estimation errors of $\phi^{G}$ in experiment: (**a**) Estimation errors of $\phi_{x}^{G}$; (**b**) Estimation errors of $\phi_{y}^{G}$; (**c**) Estimation errors of $\phi_{z}^{G}$.

The fine alignment uses the results of coarse alignment as the initial misalignment angle. To simplify the expression, the polar fine alignment algorithm proposed in this paper is defined as Algorithm 1. The conventional fine alignment algorithm is defined as Algorithm 2. And the transversal fine alignment algorithm is defined as Algorithm 3. The experiment results of fine alignment comparison are shown as Figure 6 and Table 6. As shown in Figure 6, the polar fine alignment algorithm proposed in this paper performs better than the conventional and transversal fine alignment algorithms for polar UUV navigation. The estimation errors of misalignment angles in polar fine alignment algorithm converge rapidly and they are stable near zero.

Table 6 expresses the statistical results of fine alignment in experiment.

As shown in Table 6, the *δφ_x_*, *δφ_y_* and *δφ_z_* of the polar fine alignment algorithm proposed in this paper are 0.0064′, 0.0047′ and 0.0926′, respectively. The *δφ_x_*, *δφ_y_* and *δφ_z_* of the conventional fine alignment algorithm for polar UUV navigation are 0.0334′, 0.0130′ and 14.0349′, respectively. The *δφ_x_*, *δφ_y_* and *δφ_z_* of the transversal fine alignment algorithm for polar UUV navigation are 0.2195′, 1.3639′ and 0.3468′, respectively. Performance of polar fine alignment algorithm proposed in this paper is much better than that of conventional and transversal fine alignment algorithms for polar UUV navigation.

## 6. Discussion

The simulation results and experiment results demonstrate that the proposed polar initial alignment algorithm proposed in this paper is much superior to the conventional and transversal initial alignment algorithm for polar UUV navigation. The problems in conventional initial alignment algorithm causing that it to be invalid in the polar region, the main error sources of polar coarse alignment algorithm and the main error sources of polar fine alignment algorithm are discussed as follows.

### 6.1. The Problems in Conventional Initial Alignment Algorithm Causing that It Is Invalid

In conventional initial alignment algorithm, *g* frame is chosen as the navigation frame. Due to the Earth meridians convergence rapidly in high latitude areas, conventional initial alignment algorithm has difficulties for it using in the polar region. The instruction angular velocity in SINS based on *g* frame can be described as:

(47) $$\;\omega_{en}^{n}=\left[\begin{array}{l} \omega_{enE}^{n}\\ \omega_{enN}^{n}\\ \omega_{enU}^{n} \end{array}\right]=\left[\begin{array}{c} -v_{N}/R_{Mh}\\ v_{E}/R_{Nh}\\ \left(v_{E}/R_{Nh}\right)\tan L \end{array}\right].$$

Based on Equation (47), the up directional instruction angular velocity error can be expressed as:

(48) $$\;\delta \omega_{enU}^{n}=\left(\delta v_{E}/R_{Nh}\right)\tan L,$$

**Figure 7:**
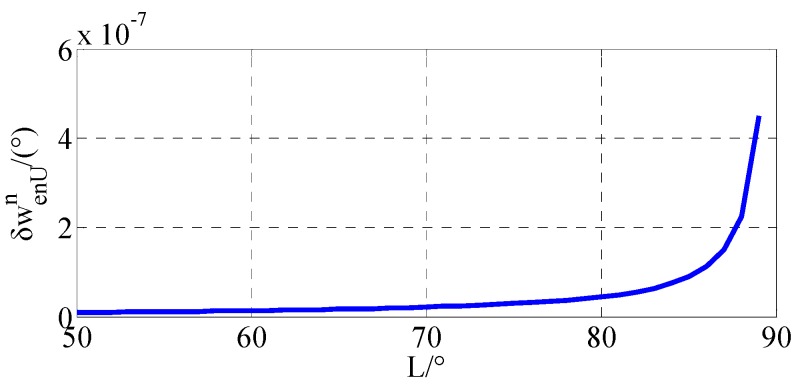
Relationship between $\delta \omega_{enU}^{n}$ and L.

Assuming that the east velocity error in *g* frame is 0.05 m/s, the relationship between $\delta \omega_{enU}^{n}$ and *L* can be described as in Figure 7. 

As shown in Figure 7, the error of the up directional instruction angle velocity is gradually approaching infinity with the latitude approaching 90°. Therefore, the conventional initial alignment is invalid for polar initial alignment. As the simulation results and the experiment results show, there are accuracy problems in the conventional initial alignment algorithm. It is necessary to propose the polar initial alignment algorithm to avoid this problem. Simulation and experiment results expressed in Section 5 demonstrate that the polar initial alignment algorithm for polar UUV navigation proposed in this paper is much superior to the conventional initial alignment algorithm for polar UUV navigation.

### 6.2. The Main Error Sources in Polar Coarse Alignment Algorithm

Error analyses are important to improve the accuracy of polar coarse alignment [25]. The main purpose of this section is to find the main error sources of polar coarse alignment. According to the principles of polar coarse alignment algorithm, the variation formulation of Equation (9) can be shown as:

(49) $$\;\delta C_{b}^{G}=\delta C_{e}^{G}C_{i}^{e}C_{b_{0}}^{i}C_{b}^{b_{0}}+C_{e}^{G}\delta C_{i}^{e}C_{b_{0}}^{i}C_{b}^{b_{0}}\;+C_{e}^{G}C_{i}^{e}\delta C_{b_{0}}^{i}C_{b}^{b_{0}}+C_{e}^{G}C_{i}^{e}C_{b_{0}}^{i}\delta C_{b}^{b_{0}},$$

where $\delta C_{b}^{G}$, $\delta C_{e}^{G}$, $\delta C_{i}^{e}$, $\delta C_{b_{0}}^{i}$ and $\delta C_{b}^{b_{0}}$ are the error matrix of $C_{b}^{G}$, $C_{e}^{G}$, $C_{i}^{e}$, $C_{b_{0}}^{i}$ and $C_{b}^{b_{0}}$, respectively.

Since $C_{e}^{G}$ is a matrix function of longitude and latitude, it is related to the position of UUV. This position can be obtained accurately by GPS. For the purpose of this paper, the errors of GPS are little enough to be neglected. The error of $C_{e}^{G}$ can be approximated as $\delta C_{e}^{G}=0$. Similarly, is a matrix function of time t and $\omega_{ie}$. The error of $C_{i}^{e}$ can be approximated as $\delta C_{i}^{e}=0$. Therefore, the errors of polar coarse alignment are mainly composed of $\delta C_{b_{0}}^{i}$ and $\delta C_{b}^{b_{0}}$.

Firstly, $\delta C_{b}^{b_{0}}$ is derived though the following steps. Due to the errors in actual condition, the update equation of ${\hat{C}}_{b}^{b_{0}}$ is described as:

(50) $${\dot{\hat{C}}}_{b}^{b_{0}}={\hat{C}}_{b}^{b_{0}}{\hat{\omega }}_{ib}^{b}\times ,$$

(51) $$\;{\hat{\omega }}_{ib}^{b}=\omega_{ib}^{b}+\varepsilon^{b},$$

(52) $$\delta C_{b}^{b_{0}}={\hat{C}}_{b}^{b_{0}}-C_{b}^{b_{0}},$$

(53) $$\;{\hat{C}}_{b}^{b_{0}}=\left[I-\varphi^{b_{0}}\times \right]C_{b}^{b_{0}},$$

where $\;\varepsilon^{b}$ is the gyro drifts and $\varphi^{b_{0}}\times$ is the anti-symmetric matrix of the error angle $\varphi^{b_{0}}$ between the actual $b_{0}$ frame and the ideal $b_{0}$ frame. And the differential form of Equation (51) can be expressed as:

(54) $$\delta {\dot{C}}_{b}^{b_{0}}=-{\dot{\varphi }}^{b_{0}}\times C_{b}^{b_{0}}-\varphi^{b_{0}}\times {\dot{C}}_{b}^{b_{0}},$$

By substituting Equations (50)–(53) into (54) and neglecting high-order terms, Equation (52) can be rewritten as:

(55) $${\dot{\varphi }}^{b_{0}}=\varepsilon^{b}.$$

Using integral algorithm $\varphi^{b_{0}}$ can be calculated from Equation (53):

(56) $$\varphi^{b_{0}}=\int {\dot{\varphi }}^{b_{0}}dt=\int \varepsilon^{b}dt=\varepsilon^{b}\Delta t,$$

(57) $$\;\varphi^{G}=C_{b_{0}}^{G}\varphi^{b_{0}}=C_{b_{0}}^{G}\varepsilon^{b}\Delta t.$$

Based on Equation (57), the main error source of $\delta C_{b}^{b_{0}}$ is the gyro drifts $\varepsilon^{b}$.

Similarly, the main error source of $\delta C_{b_{0}}^{i}$ can be derived based on the following equations:

(58) $${\hat{f}}^{b}=f^{b}+\delta f^{b},$$

(59) $$\;{\hat{f}}^{b_{0}}={\hat{C}}_{b}^{b_{0}}{\hat{f}}^{b}=\left[I-\varphi^{b_{0}}\times \right]C_{b}^{b_{0}}\left(f^{b}+\delta f^{b}\right)\;\approx f^{b_{0}}-\varphi^{b_{0}}\times f^{b_{0}}+\delta f^{b_{0}},$$

(60) $${\hat{C}}_{b_{0}}^{i}=G^{-1}\hat{F},$$

where ${\hat{f}}^{b}$ is the measurement of the accelerometer; G and F are the vector in actual condition based on Equation (25) and $\hat{F}=F+\delta F$.

By substituting Equation (59) into (25) neglecting the high-order terms and making matrix orthogonalization and square root series expansion, $C_{b_{0}}^{i}$ can be described as:

(61) $${\hat{C}}_{b_{0}}^{i}=\left[I-\frac{1}{2}{\left(C_{b_{0}}^{i}\delta F{\left(G^{-1}\right)}^{T}-G^{-1}\delta FC_{i}^{b_{0}}\right)}^{T}\right]C_{b_{0}}^{i}.$$

There are errors between the actual i frame and the ideal i frame which can be represented by $\varphi^{i}$:

(62) $${\hat{C}}_{b_{0}}^{i}=\left[I-\varphi^{i}\times \right]C_{b_{0}}^{i}.$$

Make comparison between Equations (61) and (62) and neglect the high-order terms. Therefore, after substitution and organization, $\varphi^{i}$ and the projection of $\varphi^{i}$ in *G* frame can be expressed as:

(63) $$\varphi^{i}=\frac{1}{2}{\left(C_{b_{0}}^{i}\delta F{\left(G^{-1}\right)}^{T}-G^{-1}\delta FC_{i}^{b_{0}}\right)}^{T},$$

(64) $$\;\varphi^{G}=\frac{1}{2}C_{e}^{G}C_{i}^{e}{\left(C_{b_{0}}^{i}\delta F{\left(G^{-1}\right)}^{T}-G^{-1}\delta FC_{i}^{b_{0}}\right)}^{T}.$$

Based on Equation (64), δF includes the accelerometer bias $\nabla^{b}$. Therefore, the main error source of $\delta C_{b}^{b_{0}}$ is the accelerometer bias $\nabla^{b}$. Therefore, based on Equations (57) and (64), the main error sources of the polar coarse alignment algorithm for polar UUV navigation are the gyro drifts $\varepsilon^{b}$ and accelerometer bias $\nabla^{b}$.

### 6.3. The Main Error Sources in Polar Fine Alignment Algorithm

The drifts of IMU including the gyro drifts and accelerometer bias are also the main error sources of the polar fine alignment algorithm for polar UUV navigation. Other error sources affecting the alignment accuracy of polar fine alignment are the error sources in OCTANS.

The OCTANS is composed of three fiber-optic gyroscopes and three quartz accelerometers. It is an all-in-one gyrocompass and motion sensor. The error caused by inertial sensitive element in OCTANS is mainly the gyro drift. The error of accelerometer is zero bias whose effect on the system is much smaller than that of the gyro drifts. The gyro drifts can be divided into two categories: gyro constant drifts and gyro random drifts. Gyro constant drifts belong to deterministic error and it can be eliminated by compensation. Gyro random drifts are the main error affecting the precision of the system.

## 7. Conclusions

Since the conventional initial alignment algorithm is invalid in the polar region, a polar initial alignment algorithm for polar UUV navigation is proposed in this paper to solve this navigation accuracy problem. In the polar initial alignment algorithm, the grid frame is chosen as the navigation frame. Therefore, the fast convergence of the Earth meridians has no influence on the polar initial alignment algorithm. Polar coarse alignment algorithm and polar fine alignment algorithm compose the polar initial alignment algorithm for UUV in the polar region. Simulations and experiments are conducted to compare the polar initial alignment algorithm proposed in this paper, conventional and transversal initial alignment algorithms for polar UUV navigation. The results and analyses of simulation and experiment demonstrate that the proposed polar initial alignment algorithm for UUVs is effective and accurate in the polar region.

## Figures and Tables

**Table 1:** Parameters for simulation.

Parameters	Value
Simulation time (coarse) *t_c_*/(s)	120
Simulation time (fine) *t_c_*/(s)	1200
Filtering period *H_n_*/(s)	0.01
Latitude *L*/(°)	80
Longitude *λ*/(°)	126
Amplitude of pitch/roll/yaw (°)	3/1/2
Period of pitch/roll/yaw (s)	3/5/7
Gyro constant drifts	0.03 °/h
Gyro random drifts	(0.001 °/h)^2^
Accelerometer constant bias	1 × 10^−4^ *g*_0_
Accelerometer random bias	(1 × 10^−5^ *g*_0_)^2^

**Table 2:** The simulation results of coarse alignments.

Parameters	Coarse Alignment Algorithm	Value
Mean of $\delta \phi_{x}$/(°)	Polar coarse alignment algorithm	0.5331
	Conventional coarse alignment algorithm	−3.2030
	Transversal coarse alignment algorithm	−0.5982
Mean of $\delta \phi_{y}$/(°)	Polar coarse alignment algorithm	−1.7753
	Conventional coarse alignment algorithm	2.6893
	Transversal coarse alignment algorithm	−2.1027
Mean of $\delta \phi_{z}$/(°)	Polar coarse alignment algorithm	5.9691
	Conventional coarse alignment algorithm	59.2882
	Transversal coarse alignment algorithm	6.3123
Standard deviation of $\delta \phi_{x}$	Polar coarse alignment algorithm	0.118239
	Conventional coarse alignment algorithm	0.000806
	Transversal coarse alignment algorithm	0.602804
Standard deviation of $\delta \phi_{y}$	Polar coarse alignment algorithm	0.005283
	Conventional coarse alignment algorithm	0.002867
	Transversal coarse alignment algorithm	0.502575
Standard deviation of $\delta \phi_{z}$	Polar coarse alignment algorithm	0.570818
	Conventional coarse alignment algorithm	0.000002
	Transversal coarse alignment algorithm	0.540643

**Table 3:** Estimation errors (RMS) of fine alignment algorithms in simulation.

Parameters	Fine Alignment Algorithm	Value
$\delta \phi_{x}$/(′)	Algorithm 1	0.0060
	Algorithm 2	0.0481
	Algorithm 3	0.1303
$\delta \phi_{y}$/(′)	Algorithm 1	0.0039
	Algorithm 2	0.0202
	Algorithm 3	1.2560
$\delta \phi_{z}$/(′)	Algorithm 1	0.2143
	Algorithm 2	14.8649
	Algorithm 3	0.2953

**Table 4:** The practical measured data.

Parameters	Value
Gyro constant drifts	0.03 °/h
Gyro random drifts	(4.102 × 10^−6^ rad/s)^2^
	(4.296 × 10^−6^ rad/s)^2^
	(2.375 × 10^−6^ rad/s)^2^
Accelerometer constant	1 × 10^−4^ *g*_0_
Accelerometer random bias	(0.00162 m/s^2^)^2^
	(0.002001 m/s^2^)^2^
	(0.0007122 m/s^2^)^2^

**Table 5:** The experiment results of coarse alignments.

Parameters	Coarse Alignment Algorithm	Value
Mean of $\delta \phi_{x}$/(°)	Polar coarse alignment algorithm	−0.4378
	Conventional coarse alignment algorithm	−0.1959
	Transversal coarse alignment algorithm	−0.6256
Mean of $\delta \phi_{y}$/(°)	Polar coarse alignment algorithm	−1.7750
	Conventional coarse alignment algorithm	2.5139
	Transversal coarse alignment algorithm	−2.1249
Mean of $\delta \phi_{z}$/(°)	Polar coarse alignment algorithm	5.5904
	Conventional coarse alignment algorithm	59.8336
	Transversal coarse alignment algorithm	6.3255
Standard deviation of $\delta \phi_{x}$	Polar coarse alignment algorithm	0.284479
	Conventional coarse alignment algorithm	0.276744
	Transversal coarse alignment algorithm	0.385646
Standard deviation of $\delta \phi_{y}$	Polar coarse alignment algorithm	0.279732
	Conventional coarse alignment algorithm	0.312622
	Transversal coarse alignment algorithm	0.313164
Standard deviation of $\delta \phi_{z}$	Polar coarse alignment algorithm	0.310097
	Conventional coarse alignment algorithm	0.297141
	Transversal coarse alignment algorithm	0.286084

**Table 6:** Estimation errors (RMS) of fine alignment algorithms in experiment.

Parameters	Fine Alignment Algorithm	Value
$\delta \phi_{x}$/(′)	Algorithm 1	0.0064
	Algorithm 2	0.0334
	Algorithm 3	0.2195
$\delta \phi_{y}$/(′)	Algorithm 1	0.0047
	Algorithm 2	0.0130
	Algorithm 3	1.3639
$\delta \phi_{z}$/(′)	Algorithm 1	0.0926
	Algorithm 2	14.0349
	Algorithm 3	0.3468
